# Sex Differences in Autism Spectrum Disorder: An Investigation on Core Symptoms and Psychiatric Comorbidity in Preschoolers

**DOI:** 10.3389/fnint.2020.594082

**Published:** 2021-01-28

**Authors:** Margherita Prosperi, Marco Turi, Silvia Guerrera, Eleonora Napoli, Raffaella Tancredi, Roberta Igliozzi, Fabio Apicella, Giovanni Valeri, Caterina Lattarulo, Andrea Gemma, Elisa Santocchi, Sara Calderoni, Filippo Muratori, Stefano Vicari

**Affiliations:** ^1^Department of Developmental Neuroscience, IRCCS Fondazione Stella Maris, Pisa, Italy; ^2^Department of Clinical and Experimental Medicine, University of Pisa, Pisa, Italy; ^3^Fondazione Stella Maris Mediterraneo, Potenza, Italy; ^4^Child and Adolescence Neuropsychiatry Unit, Department of Neuroscience, Bambino Gesù Children's Hospital (IRCCS), Rome, Italy; ^5^Institute of Psychiatry, Fondazione Policlinico Universitario A. Gemelli, Catholic University, Rome, Italy

**Keywords:** autism, sex differences, preschoolers, psychiatric comorbidities, child behavior checklist, autistic females

## Abstract

Findings regarding sex differences in autism spectrum disorder (ASD), as far as core symptoms and psychiatric comorbidities (PC) are concerned, are inconsistent, inconclusive, or conflicting among studies. The lower prevalence of ASD in females than in males and the age and intelligence quotient (IQ) heterogeneity among samples made it difficult to investigate these differences. This case–control study tries to deepen the impact of sex differences on core symptoms of autism and PC in 214 preschoolers with ASD (mean age, 45.26) without impairment in non-verbal IQ (nvIQ ≥70). A total of 107 ASD females (mean age, 44.51 ± 13.79 months) were matched one by one with 107 males (mean age, 46.01 ± 13.42 months) for chronological age (±6 months) and nvIQ (±6 points). We used the Autism Diagnostic Observation Schedule 2 (ADOS-2) and the Child Behavior Checklist (CBCL) 1.5–5 to explore autism severity and PC. The results highlight that ASD females did not significantly differ from ASD males regarding the severity of autism. Statistically significant lower levels of emotionally reactive (*p* = 0.005, η^2^ = 0.04), anxious-depressed (*p* = 0.001, *η*^2^ = 0.05), internalizing problems (*p* = 0.04, *η*^2^ = 0.02), and DSM-Oriented Scales anxiety problems (*p* = 0.02, *η*^2^ = 0.04) in ASD females than in ASD males were also detected. Our findings of no difference in the autism severity and lower internalizing problems in females than males with ASD extend the knowledge of autism in females during preschool years. Compared to other similar studies on this topic, we can state that these results are not supported by differences in nvIQ between sexes nor by the presence of cognitive impairment. It confirms the need for clinicians to consider sex differences when describing autism psychopathology.

## Introduction

Autism spectrum disorder (ASD) is a neurodevelopmental disorder characterized by persistent deficits in social communication and social interaction and repetitive, restricted interests or behaviors (American Psychiatric Association, 2013). Previous studies have shown a prevalence rate higher than 1% in Italy (Narzisi et al., 2018), with a male-to-female ratio close to 3:1, according to a recent systematic review and meta-analysis (Loomes et al., 2017). Although this sex imbalance remains a matter of debate, genetic, and/or sex-related hormone pathogenesis are thought to play a considerable role (Werling, 2016). Several authors had initially proposed that females diagnosed with ASD tended to have more significant autistic symptoms than males. Different biological characteristics seem to distinguish females from males with ASD (Lai et al., 2015a,b).

Identifying these biological and behavioral differences between males and females with ASD appears crucial as it influences all research on the etiology of ASD and the time of diagnosis (Little et al., 2017). For example, the higher rates of psychiatric comorbidity (PC) often reported in females with ASD have been linked to diagnosis delays.

At the brain level, it has been shown that preschoolers with ASD have sex differences. It is important to consider gender in studies examining the structural neuroanatomy of ASD since neuroimaging findings from investigations mostly or only with males may not necessarily extend to females (Retico et al., 2016). In particular, a recent study identified a close positive correlation between amygdala volume and internalizing problems in girls but not in boys with ASD (Nordahl et al., 2020), suggesting sex-specific brain–behavior relationships.

At the phenotypic level, some studies investigated the connections between sexes and clinical manifestations in ASD (Bölte et al., 2011; Andersson et al., 2013; Lai et al., 2015b; Reinhardt et al., 2015; Ratto et al., 2018; Duvall et al., 2019). Regarding the nuclear ASD symptoms, some studies have suggested the presence of sex differences in restricted and repetitive behaviors (Hartley and Sikora, 2009; Werling and Geschwind, 2013; Tillmann et al., 2018) and social communication abilities (Frazier et al., 2014; Little et al., 2017; Craig et al., 2020); in contrast, other studies showed no significant differences in autism core features between sexes (Carter et al., 2007; Andersson et al., 2013; Reinhardt et al., 2015). The wide age and IQ range of the samples and the different methodological approaches across studies may have caused these discrepant findings (Carter et al., 2007; Little et al., 2017). For example, higher-functioning girls with ASD could have a delay in diagnosis compared to males or even escape diagnosis (Lai et al., 2015b; Little et al., 2017). Besides this, young girls with ASD could have specific characteristics that parents cannot notice during everyday life. While social interaction difficulties differentiate boys with ASD from children with developmental delays and females with ASD, these are not discriminatory for females with ASD within early caregiver concerns (Little et al., 2017).

Additionally, a large number of studies showed a higher rate of PC (Lai et al., 2019; Lord et al., 2020) in males compared to females, although relatively few studies have compared sex differences in children with ASD in terms of associated PC, failing to provide consistent results. In the neurotypical population, a different prevalence ratio emerges between sexes considering DSM-5-based psychopathology according to age: in the toddler years, mental disorders are equally distributed in boys and girls (Keenan and Shaw, 1994) as well as in adolescence and adulthood (with a preponderance of internalizing problems in females) (Zahn-Waxler et al., 2008), while in preschool and elementary school, boys show more psychopathology than girls (Crick and Zahn–Waxler, 2003; Zahn-Waxler et al., 2008). In the ASD field, although there is a positive correlation between age and comorbidity (Fodstad et al., 2010), particularly for depression and anxiety (Mayes et al., 2011), discrepant findings emerge when we consider sex.

Salazar and colleagues (Salazar et al., 2015) evaluated 101 children with ASD with and without intellectual disability, with ages from 4.5 to 9.8 years, and identified female sex as a protective factor for attention-deficit hyperactivity disorder (ADHD), oppositional defiant disorder, and tic disorder. In one of the first studies on the impact of sex in high-functioning preadolescents and adolescents with ASD, Solomon et al. (2012) compared a sample of 20 girls and 20 boys with ASD, with ages from 8 to 18 years, with a group of 19 girls and 17 boys with TD and found that ASD girls are at a greater risk for internalizing problems than ASD boys and TD girls. Comparing boys and girls with high function ASD, with ages from 3 to 18 years, without differences in IQ and age range, Mandy et al. (2012) found that less externalizing problems were reported by teachers in females than in males.

A large part of previous studies used the Child Behavior Checklist (CBCL) (Achenbach and Rescorla, 2000, 2001) to investigate PC, highlighting the utility of this tool to assess associated emotional and behavioral symptoms in children and adolescents with ASD (Guerrera et al., 2019; Muratori et al., 2019). A recent systematic review observed age-related sex differences in ASD in comparison with TD peers. Regarding internalizing behaviors, early-adolescent girls with ASD showed a higher level of depressive and anxious symptoms than boys with ASD and girls with TD, whereas late-adolescent girls and boys with ASD showed no differences. In a recent study, Guerrera et al. (2019) reported lower scores in internalizing problem scales for females than males in a group of 735 children and adolescents with ASD. These findings contrast with the results of Pisula et al. (2017), who failed to find sex differences.

Concerning externalizing behaviors, ASD showed more symptoms of ADHD than TD peers. Regarding children with ASD, females with ASD also show lower ADHD scores than males (Hull et al., 2017). Frazier and colleagues (Frazier et al., 2014), analyzing the data of 2,418 children and adolescents with ASD, with ages 4–18 years, identified a higher presence of externalizing problems, such as irritability and self-injurious behavior, in females than in males. Holtmann et al. (2007), examining 23 females and 23 males matched for age, IQ, and ASD diagnoses, showed that females were more likely to experience behavioral problems, such as social, attention, and thought problems, than males.

Few studies investigated sex differences and PC in ASD preschoolers. In a review, Kirkovski et al. (2013) suggested that, although psychiatric symptoms are present at an early age, sex differences became apparent only later. Hartley and Sikora (2009) investigated the presence of associated emotional and behavior problems in ASD toddlers (range, 1.5–3.9 years) and observed more frequent sleep disorders and affective problems in females than males. A 2-year longitudinal research analyzing ASD preschoolers with ages 2–5.4 years (Postorino et al., 2015) showed lower scores on the CBCL anxiety problems subscale at baseline and lower scores on the CBCL sleep problems subscale in females than males at follow-up.

In general, studies on sex differences in PC conducted so far in children with ASD show several limitations in terms of the wide age range of the participants and the limited number of subjects involved, especially of ASD females. Moreover, the frequent inclusion in these studies of participants with a co-occurring intellectual disability does not clarify whether the behavioral disorders identified are due to the intellectual disability itself or considered a characteristic specific of ASD (Little et al., 2017). Thus, the previous investigations are not conclusive, and more studies are needed to define the impact of PC on the clinical care of children with ASD.

This study aims to explore sex differences as far as autism core symptoms and PC are concerned in a large group of ASD preschoolers without impairment in the non-verbal quotient.

## Materials and Methods

### Participants

This case–control study included 214 preschoolers, with ages between 18 and 73 months (mean age, 45.26; SD, 13.59 months), with ASD without impairment of non-verbal quotient (non-verbal IQ ≥70). A total of 107 females matched to 107 males one by one for chronological age (±6 months) and non-verbal IQ (±6 points) were examined. All the participants were selected from an initial sample of 989 preschoolers with and without non-verbal IQ impairment (mean age, 44.0 months; SD, 13.8 months; range, 16–75 months) examined in the preliminary analysis section of this study and investigated in depth in previously published research (Muratori et al., 2019).

Three different Italian care centers for children recruited enrolled patients: IRCCS Stella Maris Foundation in Pisa, IRCCS Bambino Gesù Children's Hospital in Rome, Stella Maris Mediterraneo Foundation in Matera from April 2006 to August 2018. All the enrolled subjects had a primary diagnosis of autism without other known medical or psychiatric comorbidity except for a speech disorder, a regulation disorder, and anxiety traits. Only 2.8% (six children) were foreigners and did not have Italian as their mother tongue.

The subjects' selection was implemented using a sub-sampling process without replacement, and then each observation from the dataset may appear in the sample not at all or once. The males were included in the sample only if they met the age and IQ thresholds of the female subsample, as indicated above. A custom script under Matlab R2019 allowed for the implementation of the subsampling procedure. All received a diagnosis of ASD according to DSM-5 criteria (American Psychiatric Association, 2013) or of autistic disorder, Asperger disorder, and pervasive developmental disorder—not otherwise specified according to DSM-IV criteria (American Psychiatric Association, 1994), performed by a multidisciplinary team including a senior child psychiatrist and an experienced clinically trained research child psychologist. We excluded all syndromic autism cases or known causes of ASD and children under psychotropic drugs in the last 2 months before the evaluation. All participants lived in Italy.

The current study was carried out according to the standards for good ethical practice and in accordance with the guidelines of the Declaration of Helsinki. Written informed consent from the parent/guardian of each participant was obtained when filling out the questionnaire.

### Measures

#### Autism Diagnostic Observation Scale-G and Autism Diagnostic Observation Scale-2

We used the ADOS, the gold-standard standardized interviewer-rated measure for child observation and assessment of communication skills, social interaction, quality of play, and imagination to confirm the clinical diagnosis. It consists of standardized activities that allow the examiner to observe behaviors that have been identified as relevant to the diagnosis of ASD. In this study, we used ADOS-G (Lord et al., 2000) and ADOS-2 (Lord et al., 2012). According to the two already published algorithms (Gotham et al., 2009; Hus et al., 2014), for each participant, we calculated the Calibrated Severity Score (CSS) based on ADOS total score and social affect (SA) and restricted repetitive behaviors (RRB) sub-scores. The CSS range is 1–10, and it allows comparing different versions and modules of ADOS. Moreover, the CSS provides a measure of autism symptoms independent of age and language ability and is thus better suited than the ADOS scores for assessing the severity of ASD (Shumway et al., 2012). We previously converted the scores of ADOS-G into ADOS-2 scores (SA, RRB, and total score) based on the new algorithm proposed by the algorithm of Gotham et al. (2007). The total and the CSS domains were calculated for the Toddler Module of ADOS-2 based on Esler et al. (2015) to facilitate a direct comparison with other modules of ADOS-2. Then, we used the score of item A1 of ADOS to obtain a measure of the expressive linguistic level. Thus, we have decoded the scores as follows: (0) language absent or <5 words, (1) at least five words, (2) sentences of at least three words, and (3) fluent language.

#### Cognitive Assessment

Several standardized tests were used to assess intellectual abilities due to differences in children's verbal and functioning levels. These included the Leiter International Performance Scale-Revised (LIPS- R) (Roid and Miller, 1997), the Griffiths Mental Developmental Scales-Extended-Revised (GMDS-ER) (Luiz and Knoesen, 2006), and the Italian version of Wechsler Preschool and Primary Scale of Intelligence (WPPSI) (Wechsler, 1989). When the tool provides a mental age (MA), IQ was estimated, dividing MA by the child's chronological age (CA): MA/CA × 100. For this study, we have considered the non-verbal IQ scores (performance IQ or pIQ).

#### CBCL 1½−5

The Italian version of the Child Behavior Checklist (CBCL 1½−5) (Achenbach and Rescorla, 2000; Frigerio et al., 2006) is one of the most widely used checklists consisting of 100 statements about a child's behaviors. The parents rate each behavior's frequency on a three-point Likert scale (0, not true; 1, somewhat or sometimes true; 2, very true or often true). The CBCL provides seven syndrome scale scores (i.e., emotionally reactive, anxious/depressed, somatic complaints, withdrawn, aggressive behavior, attention problems, and sleep problems), three summary scale scores (i.e., internalizing, externalizing, and total problems), five DSM-oriented scales (DOS) [i.e., affective problems, anxiety problems, pervasive developmental disorder problems (PDP), attention deficit hyperactivity disorder (ADHD) problems, and oppositional defiant problems]. A T-score of 64 and above for summary scales and a T-score of 70 and above for syndrome and DSM-oriented scales are generally considered as clinically significant. Values between 60 and 63 for summary scales or between 65 and 69 for syndrome and DSM-oriented scales identify a borderline clinical range. Values below 60 for the summary scales or below 65 for the other scales are not clinically significant. Each scale of the DOS and syndromic scales is independent of each other because it is composed of independent items (e.g., item 98 “withdrawn” is represented only in the PDP scale of the DOS and withdrawn scale of the syndromic scales). CBCL syndrome scales are sensitive to measuring general mental illness/psychopathology (Jensen et al., 1993; Kasius et al., 1997); DOS are instead used to measure specific DSM diagnoses.

In this study, we adopted the borderline clinical elevation cutoff score (*T* score ≥60 for summary scales and *T* score ≥65 for DOS), according to previous studies on screening (Muratori et al., 2011; Narzisi et al., 2013; Rescorla et al., 2015) and PC (Llanes et al., 2018; Muratori et al., 2019) in young children with ASD.

### Procedure

All participants received a clinical diagnosis of ASD and were assessed with ADOS and a proper psychometric test. We did not include children who were not evaluable with the standardized tests due to behavior problems.

The parents completed the CBCL at the beginning of the diagnostic process, based on their child's behavior in the current status or the past 2 months. For this study, the CBCL completed by mothers was preferred; when this was not possible, the CBCL was completed by fathers or by another close caregiver.

Firstly, we conducted a preliminary analysis on the whole sample of 989 preschoolers with ASD, thus also including the participants with non-verbal IQ <70, to define gender differences in clinical characteristics and PC in males vs. females. This pre-analysis allowed us to consider both the influence of autism and intellectual disability on gender differences, given the frequent coexistence of these two conditions.

Then, we examined the subsample of 214 subjects by comparing males to females on the clinical measures used in this study and examining PC differences. In this way, only the DSA could be supposed to have an impact on gender differences. Subsequently, we looked for sex differences in clinical variables comparing males vs. females based on no, single, or multiple PC. Finally, we studied the possible correlations between the severity of autism measured through the CSS-Total Score and some scales of the CBCL both in males and females.

## Data Analysis

All the continuous variables were examined for normality using skewness tests and Kolgomorov–Smirnov testing. Descriptive analyses, chi-square analysis, and *t*-tests were used for categorical and continuous independent variables, respectively. One-way analysis of variance (ANOVA) was performed to evaluate differences in age, performance IQ, and CBCL scales among all groups. Two-way ANOVA with Bonferroni *post-hoc* test for multiple comparisons was used to assess sex differences in performance IQ and ADOS scores among the different comorbidity groups.

To evaluate effect size, we measured:

- Cohen's d (*d*) for independent sample *t*-test,
- eta squared (*η*^2^ that represents the variance accounted for) for analysis of variance, and
- Phi (*ϕ*) for non-parametric statistics (chi-square).

Simple bivariate Pearson correlations were used to assess the possible association between autism severity and CBCL scales across genders.

## Results

### Preliminary Analysis

Considering the whole sample, we found a statistically significant difference in RRB-CSS, where males showed higher mean scores than females with ASD. No significant differences were found for age, non-verbal IQ, CSS-Total Score, and CSS-SA. The males and females did not show any difference in mean DOS score over the borderline cutoff (Table 1). Males had higher mean scores in the anxiety problems than females.

**Table 1:** The clinical differences in age, performance IQ, ADOS CSS, PDP scale of the CBCL, and PC between male and female subjects in the whole sample (*n* = 989).

		**Male**	**Female**	***t*-test**	***p*-value**	**Effect size**
Age		44.08 (13.92)	43.67 (13.06)	0.32	0.74	–
Performance IQ		76.68 (23.17)	76.58 (23.82)	1.61	0.10	–
ADOS CSS-SA		6.11 (02.16)	6.15 (1.92)	−0.20	0.84	–
ADOS CSS-RRB		*7.02* *(2.04)*	*6.57* *(2.16)*	*2.64*	*0.008*	*d = 0.21*
ADOS CSS-Total Score		6.27 (1.92)	6.20 (2.01)	0.48	0.62	–
CBCL-PDP		68.89 (9.80)	67.44 (9.15)	1.75	0.08	–
Affective problems	A	58.95 (8.86)	58.21 (8.36)	0.99	0.32	–
	B	23.5	22.5	0.08	0.76	–
Anxiety problems	A	*56.98* *(8.30)*	*55.97* *(7.13)*	*2.06*	*0.04*	*d = 0.09*
	B	17.6	12.4	2.65	0.10	–
Attention deficit/hyperactivity problems	A	57.75 (6.89)	58.05 (7.33)	−0.50	0.61	–
	B	17.0	18.9	0.37	0.53	–
Oppositional problems	A	54.50 (6.11)	54.59 (6.02)	−0.14	0.88	–
	B	7.9	7.7	0.01	0.91	–

*Significant comparisons are highlighted in italics*.

*A, mean and SD; B, number of subjects in the borderline/clinical range expressed as a percentage of males or females; ADOS, Autism Diagnostic Observation Schedule; CBCL-PDP, Child Behavior CheckList-Pervasive Developmental Problems; CSS, Calibrated Severity Score; IQ, intelligence quotient; M, mean; RRB, restricted and repetitive behaviors; SA, social affect; SD, standard deviation*.

### Children With ASD Without Impairment of the Non-verbal IQ

No significant difference was present for mean age [*F*_(213)_ = 0.76, *p* = 0.42] and performance IQ [*F*_(213)_ = 0.10, *p* = 0.98] between males and females, as shown in Table 2. The distribution of expressive language level, measured through the ADOS Score on item A1, revealed an association between gender and language level (*X*^2^ = 11.13, *p* = 0.01). In particular, we found a higher percentage of absent language in females (64.5%) compared with the males (42.1%).

**Table 2:** Age, performance IQ, ADOS CSS, PDP scale of the CBCL, and language in children with ASD without impairment of the non-verbal IQ (*n* = 214) and clinical differences between male (*n* = 107) and female (*n* = 107) subjects.

	**Whole** **sample** **(*n* = 214)**	**Male** **(*n* = 107)** ***M* (SD)**	**Female** **(*n* = 107)** ***M* (SD)**	***t*-test** ***X***^**2**^	***p*-value**
Age	45.26 (13.59)	46.01 (13.42)	44.51 (13.79)	0.80	0.42
Performance IQ	90.88 (14.40)	90.90 (14.00)	90.87 (14.86)	0.01	0.98
ADOS CSS-SA	5.68 (1.78)	5.79 (1.80)	5.56 (1.76)	0.95	0.34
ADOS CSS-RRB	6.55 (1.99)	6.68 (1.91)	6.41 (2.07)	0.99	0.32
ADOS CSS-Total Score	5.78 (1.87)	5.85 (1.90)	5.71 (1.85)	0.54	0.58
CBCL-PDP	67.00 (10.12)	67.99 (11.17)	66.00 (8.88)	1.44	0.15
Language (0) (1) (2) (3)	*53.3% (114)* *30.8% (66)* *14% (30)* *1.9% (4)*	*42.1% (45)* *38.3% (41)* *16.8% (18)* *2.8% (3)*	*64.5% (69)* *23.5% (25)* *11.2% (12)* *0.9% (1)*	*11.13*	*0.01*

*Significant comparisons are highlighted in italics*.

ADOS, Autism Diagnostic Observation Schedule; CBCL-PDP, Child Behavior CheckList-Pervasive Developmental Problems; CSS, Calibrated Severity Score; IQ, intelligence quotient;

*M, mean; RRB, restricted and repetitive behaviors; SA, social affect; SD, standard deviation*.

#### Autism Core Symptoms

We compared males and females in the CSS scores of the ADOS. No difference in CSS-SA, CSS-Total Score, and CBCL-PDP scale were found.

#### Psychiatric Comorbidity

As far as CBCL syndrome scales (Table 3) are concerned, males had significantly higher mean *T*-scores in the emotionally reactive (*p* = 0.005, *η*^2^ = 0.04) and anxious-depressed (*p* = 0.001, *η*^2^ = 0.05) scales than females. In all these scales, the males' rates over borderline scores were significantly higher than the females' rates (emotionally reactive: *p* = 0.02, *ϕ* = 0.15, OR = 2.1; anxious-depressed: *p* = 0.005, *ϕ* = 0.19, OR = 3.14). Regarding summary scales, males had significantly higher mean *T*-scores on internalizing problems (*p* = 0.04, *η*^2^ = 0.02) than females. The rates of males over borderline scores were significantly higher than the females' rates on total problems (*p* = 0.04, *ϕ* = 0.14, OR = 1.84).

**Table 3:** Differences in syndrome and summary scales of CBCL broad-band between male and female children with autism spectrum disorder without impairment of the non-verbal IQ.

**Behavior symptoms**		**Male**	**Female**	***F*-values** **or** ***X***^**2**^	***p*-value**	**Effect size** **(OR)**
Emotionally reactive	A	*58.55 (8.86)*	*55.52 (6.68)*	*7.96*	*0.005*	*0.04*
	B	*26.9*	*16.8*	*5.16*	*0.02*	*Φ = 0.15* *OR = 2.1*
Anxious depressed	A	*57.55 (7.83)*	*54.20 (5.55)*	*12.46*	*0.001*	*0.05*
	B	*22.4*	*8.4*	*8.06*	*0.005*	*Φ = 0.19* *OR = 3.14*
Somatic complaints	A	57.35 (8.00)	55.88 (7.26)	1.97	0.16	–
	B	23.4	15.0	2.44	0.11	–
Withdrawn	A	68.14 (10.33)	66.55 (9.92)	1.34	0.24	–
	B	62.6	54.2	1.55	0.21	–
Sleep problems	A	54.99 (7.22)	55.21 (7.77)	0.05	0.26	–
	B	5.6	8.4	0.64	0.42	–
Attention problems	A	60.86 (8.45)	59.80 (8.25)	0.0.85	0.35	–
	B	36.4	29.9	1.03	0.31	
Aggressive behavior	A	55.24 (6.58)	54.25 (6.17)	1.28	0.25	–
	B	10.3	7.5	0.52	0.47	–
Internalizing problems	A	*59.85 (11.58)*	*56.79 (10.73)*	*4.03*	*0.04*	*0.02*
	B	47.7	39.3	01.54	0.21	–
Externalizing problems	A	54.06 (10.46)	52.87 (9.56)	0.75	0.38	–
	B	26.2	15.9	3.40	0.06	***–***
Total problems	A	58.21 (11.63)	55.44 (9.79)	3.56	0.06	–
	B	*38.3*	*25.2*	*4.22*	*0.04*	*Φ = 0.14* *OR = 1.84*

*Significant comparisons are highlighted in italics*.

*A, mean and SD; B, number of subjects in the borderline/clinical range expressed as a percentage of male or female subjects*.

As far as DOS (Table 4) is concerned, males had significantly higher mean *T*-scores on anxiety problems (*p* = 0.02, *η*^2^ = 0.04) than females; the percentages of males over borderline scores in this scale were statistically significantly higher than in females (*p* = 0.001, *ϕ* = 0.2, OR = 3.43).

**Table 4:** Differences in DSM-Oriented Subscale of CBCL broad-band between male and female children with autism spectrum disorder without impairment of the non-verbal IQ.

**Psychiatric comorbidities**		**Male**	**Female**	***F*-values** **or** ***X***^**2**^	***p*-value**	**Effect size** **(OR)**
Affective problems	A	59.00 (9.03)	57.01 (7.39)	3.11	0.07	–
	B	25.2	18.7	1.33	0.24	–
Anxiety problems	A	*58.26 (8.84)*	*54.92 (6.81)*	*9.60*	*0.02*	*0.04*
	B	*26.2*	*9.3*	*10.36*	*0.001*	*Φ = 0.2* *OR = 3.43*
Attention deficit problems	A	57.16 (6.56)	56.91 (7.25)	0.07	0.79	–
	B	15.00	17.8	0.30	0.57	–
Oppositional problems	A	54.34 (6.04)	53.62 (5.47)	0.83	0.36	–
	B	7.5	5.6	0.30	0.58	–

*Significant comparisons are highlighted in italics*.

*A, mean and SD; B, number of subjects in the borderline/clinical range expressed as a percentage of male or female subjects*.

**Figure 1:**
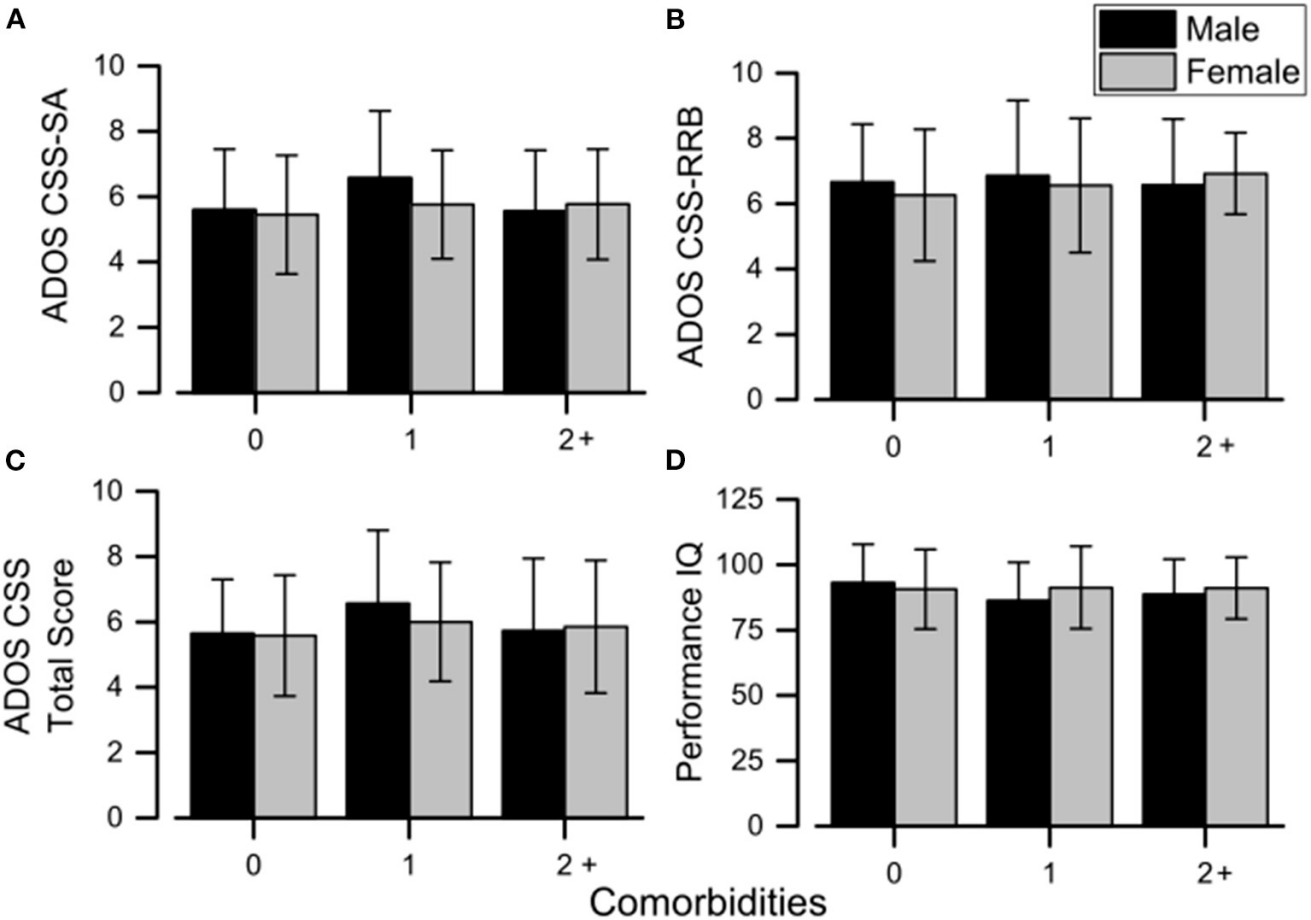
Mean sex differences in ADOS scores **(A–C)** and performance IQ **(D)** across all the comorbidity groups. Error bars correspond to ±1 SEM. ADOS, Autism Diagnostic Observation Schedule; CSS, calibrated severity score; IQ, intelligence quotient; RRB, restricted and repetitive behaviors; SA, social affect.

Considering CBCL-DOS (Table 5), 41.0% (44/107) of males and 35.5% (38/107) of females had a score over the borderline cutoff on one or more of the DOS. It means that these children (82/214) had at least one PC in addition to ASD. No differences emerged in performance IQ and CSS scores in the comparison between males and females when also considering the absence of other PC (all *p* > 0.05) or one another PC (all *p* > 0.05) or two or more PC (all *p* > 0.05) in addition to ASD. (as showed in Figure 1).

**Table 5:** Differences in ADOS scores and performance IQ (means and SD) across all comorbidity groups.

	**Comorbidities**											
	**0** ***(N*** **=** ***132*****)**			**1** ***(N*** **=** ***46)***			**2+** ***(N*** **=** ***36)***			**ANOVA** **(main effects)**		
	**Male** **(*n* = 63)**	**Female** **(*n* = 69)**	**Whole** **sample**	**Male** **(*n* = 21)**	**Female** **(*n* = 25)**	**Whole** **sample**	**Male** **(*n* = 23)**	**Female** **(*n* = 13)**	**Whole** **sample**	**Comorbidities** ***F*-values** ***p*-value**	**Gender** ***F*-values** ***p*-value**	**Interaction** ***F*-values** ***p*-value**
ADOS CSS-SA	5.59 (1.75)	5.45 (1.82)	5.52 (1.78)	6.57 (2.06)	5.76 (1.66)	6.13 (1.87)	5.65 (1.155)	5.77 (1.69)	5.69 (1.58)	*F*_(2,208)_ = 2.25 *p* = 0.10	*F*_(1,208)_ = 0.91 *p* = 0.33	*F*_(2,208)_ = 0.81 *p* = 0.44
ADOS CSS-RRB	6.67 (1.76)	6.26 (2.02)	6.45 (2.00)	6.86 (2.30)	6.56 (2.06)	6.70 (2.15)	6.57 (2.02)	6.92 (1.25)	6.69 (1.77)	*F*_(2,208)_ = 0.41 *p* = 0.66	*F*_(1,208)_ = 0.12 *p* = 0.72	*F*_(2,208)_ = 0.48 *p* = 0.61
ADOS CSS-Total Score	5.65 (1.65)	5.58 (1.85)	5.61 (1.75)	6.57 (2.24)	6.00 (1.82)	6.26 (2.02)	5.74 (2.21)	5.85 (2.03)	5.78 (2.05)	*F*_(2,208)_ = 2.216 *p* = 0.11	*F*_(1,208)_ = 0.34 *p* = 0.56	*F*_(2,208)_ = 0.39 *p* = 0.67
Performance IQ	93.18 (14.70)	90.67 (15.25)	91.87 (12.56)	86.38 (12.56)	91.32 (15.71)	89.07 (14.42)	88.74 (12.34)	91.08 (11.76)	89.58 (12.02)	*F*_(2,208)_ = 0.87 *p* = 0.40	*F*_(1,208)_ = 0.45 *p* = 0.50	*F*_(2,208)_ = 1.27 *p* = 2.08

*Results fr om the two-way ANOVA are reported and highlighted in bold when significant*.

*ADOS, Autism Diagnostic Observation Schedule; CSS, Calibrated Severity Score; IQ, intelligence quotient; M, mean; RRB, restricted and repetitive behaviors; SA, social affect*.

A bivariate Pearson correlation (to investigate the possible association between the severity of autism, measured through the CSS-Total Score, and externalizing, internalizing, and total problems scales of the CBCL separated by sex) revealed a significant correlation between CSS-Total Score and the internalizing problems scale in females [females *r*_(107)_ = 0.228, *p* = 0.01) (Table 6).

**Table 6:** Pearson correlations among autism severity and the CBCL Summary Scales are shown.

	**Males (107)**			
**Females (107)**	**ADOS** **CSS-Total Score**	**Internalizing** **problems**	**Externalizing** **problems**	**Total** **problems**
**ADOS CSS-Total Score**		0.09 (0.34)	0.04 (0.62)	0.082 (0.40)
**Internalizing problems**	*0.228* *(0.010)*		*0.624* *(0.000)*	*0.864* *(0.000)*
**Externalizing problems**	−0.046 (0.640)	*0.620* *(0.000)*		*0.825* *(0.000)*
**Total problems**	0.107 (0.275)	*0.835* *(0.000)*	*0.889* *(0.000)*	

*Bivariate Pearson correlation coefficients and p-value are shown above the diagonal for the male group (n = 107) and below the diagonal for the female group (n = 107)*.

*ADOS, Autism Diagnostic Observation Schedule; CSS-Total Score, Calibrated Severity Score-Total Score*.

## Discussion

To our knowledge, this is the first study to examine sex differences in autism symptoms and PC in a broad and homogeneous sample of ASD pre-schoolers without non-verbal intellectual impairment.

In this sample, females and males with ASD did not differ in terms of the severity of autism. This finding is not in agreement with other investigations (Carter et al., 2007; Hartley and Sikora, 2009; Frazier et al., 2014) in which females had less restricted, repetitive patterns of behavior, interests, or activities and/or more significant social communication difficulties than males with ASD. Interestingly, in a study explicitly examining if early caregiver concerns differentiated girls with ASD, boys with ASD were more likely to have social interaction concerns than girls with ASD (Little et al., 2017). It is of note that the authors did not consider the role of developmental quotient (they studied a community-based sample).

Our results are consistent *vice versa* with some recent studies (Carter et al., 2007; Holtmann et al., 2007; Banach et al., 2008) and with a systematic review and meta-analysis on sex and age differences in the core symptoms of ASD (Van Wijngaarden-Cremers et al., 2014). In the study of Van Wijngaarden-Cremers et al., ASD children below 6 years old were the only subgroup who showed no sex differences in social, communication, and repetitive and stereotyped behaviors. In contrast, older male children, male adolescents, and male adults with ASD displayed a more repetitive and stereotyped behavior than their female peers.

The investigation on the whole sample of 989 children without a selection based on non-verbal IQ allowed us to suppose that the identified RRB's gender differences were possibly due to intellectual disability rather than ASD. In fact, the analogous analysis on the restricted sample corrected for IQ did not reveal this difference between males and females. There is evidence that the RRB in ASD may be mediated by the cognitive ability level (Bodfish et al., 2000; Bishop et al., 2006; Richler et al., 2010). These results followed our previous investigation (Fulceri et al., 2016) on a different sample of preschoolers with ASD: we did not detect differences of RRB between males and females matched for non-verbal IQ. Other authors found no gender difference in RRB (Andersson et al., 2013; Joseph et al., 2013), and *vice versa*, others observed milder RRB among females compared to males with ASD (Hartley and Sikora, 2009; Sipes et al., 2011; Mandy et al., 2012), but not all those reports have considered nvIQ.

Concerning PC, we found significantly higher internalizing problems in males than in females with ASD. Studies on internalizing symptoms have produced different findings (Mandy et al., 2012; Nasca et al., 2019), and to our knowledge, this is the first study to indicate more internalizing symptom scores for males than females in a sample of ASD pre-schoolers without impairment in performance IQ. In a large sample of children and adolescents with ASD (with ages from 2.6 to 17.8 years) in which the authors investigated comorbidities through the CBCL, but without a selection of the sample based on developmental quotient or performance IQ (Guerrera et al., 2019), similar results emerged, with males exhibiting more internalizing problems than females.

Conversely, higher parent-rated anxiety (Solomon et al., 2012; May et al., 2014) and depression (Oswald et al., 2016) were detected in ASD females than in ASD males during adolescence. Adolescence could be a phase of particular vulnerability for females with ASD due to a unique combination of genetic, hormonal, and psychosocial stress, possibly causing higher rates of internalizing problems compared to ASD males in this specific period (Oswald et al., 2016).

Considering DOS, our findings reveal higher scores and higher rates of anxiety problems in ASD males than in ASD females. Our findings were in contrast with most studies reporting no influence of sex on anxiety symptoms in ASD (see Vasa and Mazurek, 2015 for a recent review). Studies examining preschool mental health argue that these years represent a crucial period for the individual in the transition from engaging in solitary or parallel play to more complex, interactive group play and in acquiring more skills in empathizing with others and in understanding the needs and emotions of peers (Bufferd et al., 2016). The failure to experience these situations and develop these social skills could increase anxiety symptoms by causing a vicious cycle. We speculate that the female sex may represent a protective factor in developing an anxiety disorder in autism: these abilities that are stressed during the preschool age may emerge with even more difficulty in males than in females precisely due to sex-specific characteristics. Investigations examining whether greater ASD severity and higher IQ place individuals with ASD at an increased risk for depression and anxiety have provided mixed results; the best combined predictors of depression and anxiety were a higher ASD severity, verbal IQ, and age, explaining close to 25% of the variance (Mayes and Calhoun, 2011).

Having studied these comorbidities both in the enlarged sample regardless of nvQI and in the restricted sample controlled for nvIQ allowed us again to hypothesize that autism in preschooler males is characterized by a higher incidence of anxiety problems than in females. Specifically, the matching between sexes based on nvIQ did not delete the significant difference with higher mean scores in males than in females and, besides that, allowed us to identify significantly higher percentages of the males over borderline scores than females in this scale.

To explore the direct correlation between ASD severity and internalizing problems, we analyzed correlations between some scales of the CBCL and CSS-Total Scores in males and females. While the association between internalizing, externalizing, and total problems in both sexes is not surprising due to the construct of the CBCL itself, it is interesting to note the additional association between ASD severity and internalizing problems specific only for females with ASD.

The rates of subjects with at least one psychiatric disorder plus ASD were similar in males and females and were much lower than previously found in older ASD subjects (Leyfer et al., 2006; Simonoff et al., 2008). The lower age and the limited range of PC explored by CBCL could partly explain this finding in our sample. Moreover, these two studies also included subjects with low skill levels. We cannot exclude the fact that this choice may have increased multiple PC in their samples since intellectual disability and developmental impairments represent a known risk factor for psychiatric disorders (Matson and Williams, 2013).

Considering the same number of PCs, males and females showed no significant differences in the severity of autism and performance IQ. Therefore, it is possible to exclude that a different distribution of comorbidities within males and females may have overshadowed the sex differences regarding the autism and developmental profile characteristics in the whole sample. On the other hand, we can exclude that the sex or number of comorbidities could influence the severity of autism or non-verbal IQ impairment.

We did not find higher rates of externalizing problems in males than in females, in contrast to the findings of Mandy et al. (2012), Frazier et al. (2014), and similarly with other studies (Brereton et al., 2006; Postorino et al., 2015). The children enrolled in the study of Mandy et al. (2012) were older, and the range of ages was wider, although the performance IQ was similar (≥71.8 in all subjects) to that of ours. In the study by Brereton et al. (2006), the age range considered was wide, and intellectual disability was present in more than 70% of the participants. In the longitudinal study of Postorino et al. (2015), although all enrolled children with ASD were preschoolers as in our sample, females showed significantly lower developmental quotients than males at baseline assessment. In the study by Frazier et al. (2014), the ASD females showed significantly lower non-verbal cognitive scores than males, and there was a lower proportion of females with IQ ≥80 relative to IQ <80 (*p* < 0.001). A meta-analysis that will critically analyze the data considering the influence of age and, particularly, of IQ on sex differences in ASD psychopathology could decrease these results' heterogeneity.

For example, we did not find significant differences in the rate of attention problems between males and females; this result contrasts with that of Holtmann et al. (2007), who detected more significant attention difficulties in ASD female samples with higher functioning than in ASD males. ADHD is one of the most common coexisting disorders in children with ASD, and it is more frequent in males than in females (Giarelli et al., 2010; May et al., 2016), especially in association with intellectual disability and during preschool age (Davis and Kollins, 2012).

The wide and variable range of cognitive levels among participants, the differences in the baseline characteristics between males and females, and the wide age ranges considered across the studies may have masked or biased the sex differences in externalizing problems (Lai et al., 2011; Mandy et al., 2012).

## Limitations

First of all, the PC has been evaluated through the parent-report questionnaires' administration and completed by only one parent. Studies that analyze reports from both parents may be more informative, and the lack of a direct clinical examination and the limited range of psychopathology explored by CBCL could limit our results. However, the efficacy of the CBCL to evaluate the presence of co-occurring emotional and behavioral disorders in ASD pre-schoolers has been widely acknowledged (Pandolfi et al., 2009; Mcconachie et al., 2015), as well as its proven construct validity (Pandolfi et al., 2012, 2014). The difficulty to directly assess the presence of psychopathology in this specific population composed of ASD pre-schoolers (Mannion et al., 2014; Matson, 2015; Cicchetti, 2016) should be noted: indeed the recognition and the evaluation of psychiatric disorders in subjects with communication difficulties could be particularly challenging (Bryson and Smith, 1998). Excellent convergence has also been reported between clinically diagnosed ADHD, conduct disorder, depression, and anxiety disorder and the related subscales of the CBCL (Biederman et al., 1993; Magyar and Pandolfi, 2017) and, more broadly, of CBCL broad-band scales with structured diagnostic interviews within psychopathology investigations (Petty et al., 2008; Hallerod et al., 2010). Besides that, a sex-specific profile can be outlined with this tool according to the female or male sex.

A second limitation is that we have to consider the parental stress index's impact on the child's parental report. For example, a parent who is particularly frustrated by his child's behavior can overestimate the symptoms or, *vice versa*, can underestimate them because of his/her difficulty in accepting his/her child's condition. We cannot exclude the fact that higher stress levels mediate the higher scores obtained by male children in their caregivers.

Moreover, we have no longitudinal data about the evolution of associated psychopathology retrospectively studied. Therefore, we cannot extend these results outside the specific range of ages examined in our ASD sample, and a cause-and-effect relationship cannot be evaluated. The relative novelty of exploring sex differences in autism and CP symptoms in a large and homogeneous sample of preschool children with ASD without non-verbal intellectual impairment did not allow us to have an *a priori* idea of our analyses based on literature results. For this reason, our analyses may appear characterized by a highly exploratory nature.

Finally, we did not include a TD control group. Therefore, we cannot exclude that our observed sex differences in behavioral symptoms characterize ASD or reflect those found in the general population (Hull et al., 2017).

## Conclusions

In conclusion, the ASD phenotype's characterization in females is a crucial issue since previous studies argued that sex differences could lead to a delayed, wrong, or missed diagnosis in girls (and women) with autism (Rivet and Matson, 2011). Previously, contradictory or inconsistent results depending on the criteria adopted for a comorbid diagnosis [e.g., an additional diagnosis of ADHD in subjects with ASD was not allowed in the DSM-IV and the DSM-IV-TR (American Psychiatric Association, 1994)], the inclusion of cognitively/functionally different subjects (Lai et al., 2011; Mandy et al., 2012), the broad age ranges examined (Worley and Matson, 2011), and the lack of quite large samples of females (Nasca et al., 2019) have been reported, possibly obscuring the ASD female phenotype and sex differences in ASD.

In light of this, our results have contributed to characterizing ASD females compared to ASD males in autistic symptoms and PC, regardless of age and IQ confounding variables.

It could be useful in future studies to include a parenting stress assessment with a self-report to evaluate the level of stress specifically related to the parental role, e.g., the Parenting Stress Index-Short Form (PSI-SF) (Haskett et al., 2006). This index is frequently used in parents of autistic children, and it seems to be associated with disruptive behavior, higher levels of social disability, and lower levels of adaptive functioning of children (Postorino et al., 2019).

## Data Availability Statement

The raw data supporting the conclusions of this article will be made available by the authors, without undue reservation.

## Ethics Statement

The studies involving human participants were reviewed and approved by IRCCS Stella Maris committee. Written informed consent to participate in this study was provided by the participants' legal guardian/next of kin.

## Author Contributions

MP, MT, SG, and EN participated in the work design and wrote the manuscript's first draft. MT analyzed the data. MP, MT, SG, EN, RI, and FA evaluated the patients and collected the data. RT, GV, CL, AG, ES, SC, FM, and SV helped evaluate, edit the manuscript, and perform a critical revision. All authors has seen and approved the submission of this manuscript's version and takes full responsibility of the manuscript.

## Conflict of Interest

The authors declare that the research was conducted in the absence of any commercial or financial relationships that could be construed as a potential conflict of interest.

## Abbreviations

CBCL: Child Behavior CheckList
CSS: Calibrated Severity Score
IQ: intelligent quotient
M: mean
PDP: pervasive developmental problems
RRB: restricted and repetitive behaviors
SA: social affect
SD: standard deviation.
